# Interstitial Lung Disease and Pulmonary Damage in Primary Sjögren’s Syndrome: A Systematic Review and Meta-Analysis

**DOI:** 10.3390/jcm12072586

**Published:** 2023-03-29

**Authors:** Onorina Berardicurti, Annalisa Marino, Irene Genovali, Luca Navarini, Settimio D’Andrea, Damiano Currado, Amelia Rigon, Luisa Arcarese, Marta Vadacca, Roberto Giacomelli

**Affiliations:** 1 Clinical and Research Section of Rheumatology and Clinical Immunology, Fondazione Policlinico Universitario Campus Bio-Medico, Via Alvaro del Portillo 200, 00128 Rome, Italy; 2 Rheumatology and Clinical Immunology, Department of Medicine, School of Medicine, University of Rome “Campus Bio-Medico”, 00128 Rome, Italy; 3 Endocrinology Outpatient Clinic, ASL Avezzano-Sulmona-L’Aquila, 67039 Sulmona, Italy

**Keywords:** Sjogren’s syndrome, pulmonary involvement, interstitial lung disease, UIP, NSIP, meta-analysis, HRCT

## Abstract

Background: Pulmonary lung involvement is the most common extra-glandular manifestation in patients with primary Sjögren’s syndrome (pSS), leading to a worsening of the patient’s prognosis. To date, different studies have assessed the prevalence of pulmonary involvement and interstitial lung disease (ILD) in pSS patients with different results. Methods: We performed a systematic literature review and meta-analysis on ILD pooled prevalence in pSS according to the PRISMA and MOOSE guidelines. Furthermore, we explored the pooled prevalence of the two main presentations of pSS-ILD, nonspecific interstitial pneumonia (NSIP) and usual interstitial pneumonia (UIP). Results: We analysed the pSS-ILD prevalence in 30 studies including 8255 pSS patients. The pSS-ILD pooled prevalence was 23% (95% CI: 16–30). For NSIP, we found a pooled prevalence of 52% (CI 41–64), and for UIP we found a pooled prevalence of 44% (CI: 32–55). Regarding the meta-regression analysis, male gender, DLco value, country, and HRCT seem to contribute to the ILD presence. Conclusions: At least 20% of pSS patients have a comorbid ILD, usually NSIP. Male gender and alteration in DLco value may be considered the most important independent factors supporting an active search of lung complications during the clinical history of pSS patients.

## 1. Introduction

Sjögren’s syndrome (pSS) is a systemic autoimmune disease primarily affecting the exocrine glands with lymphocytic infiltrations, leading to their loss of function and the dryness of major mucosal surfaces, and eventually involving several internal organs [1]. When present, the internal organs’ involvement dramatically influences the clinical course of the disease and the prognosis of pSS patients [2]. Pulmonary manifestations are the most prevalent extra-glandular complications, often subclinical and difficult to assess, that must be suspected when impaired respiratory function or dry cough appears [3]. Some controversial data may be found in the available literature concerning the incidence of lung involvement, its histopathologic features, and factors associated with the development of severe complications [4,5,6]. These concerns may partially explain why lung involvement in pSS patients still represents a major challenge leading to poor survival and an increase in mortality [4,5,6].

Interstitial lung disease (ILD) is the most serious pulmonary complication in pSS patients, and some reports showed that the ILD cumulative incidence in pSS was 10% 1 year after diagnosis, increasing to 20% after 5 years. ILD may be diagnosed based on clinical presentations, high-resolution computed tomography (HRCT), pulmonary function tests (PFTs) and, eventually, lung biopsy [1,5,7]. HRCT is a sensitive diagnostic tool for ILD and strongly correlates with pulmonary histology and PFTs, the most common HRCT patterns found in pSS patients being nonspecific interstitial pneumonia (NSIP), the usual interstitial pneumonia (UIP), lymphoid interstitial pneumonia, organizing pneumonia (OP), and finally bronchiolitis. A minority of patients may show an indeterminate radiologic pattern [6]. In pSS patients, different studies suggest that NSIP is the most frequent radiologic pattern observed in 41–45% of patients, followed by UIP in about 10% and OP in 4% of the patients. A combination of these patterns can be seen in up to 40% of pSS patients [7]. Many risk factors have been associated with the development of pulmonary involvement in pSS, some depending on lifestyle, others related to co-morbidities, and finally some linked to specific biologic activities of the disease, mainly related to B-cells activation, such as hypergammaglobulinemia and the presence of autoantibodies [6].

To better define the pSS-ILD pooled prevalence, to assess the HRCT’s more frequent patterns, and to identify the risk factors associated with ILD, we performed a systematic literature review (SRL) and consequently analysed all the available data deriving from many studies from the beginning of the 1980s until now.

Our results concerning pSS-ILD, deriving from all the available literature retrieved from three scientific sources, allow us to give a clearer picture of this systemic manifestation, better defining the subgroups of patients affected by ILD and, finally, filling the gaps deriving from the single studies results.

## 2. Materials and Methods

### 2.1. Protocol

This study was carried out in accordance with Cochrane Collaboration and the Preferred Reporting Items for Systematic reviews and Meta-Analyses Protocols (PRISMA-P) statement [8]. It also complies with the guidelines of Meta-Analyses and Systematic Reviews of Observational Studies (MOOSE) [9]. The PRISMA-P and MOOSE checklists have been presented as Supplementary Tables S1 and S2, respectively.

### 2.2. Search Strategy

**Figure 1:**
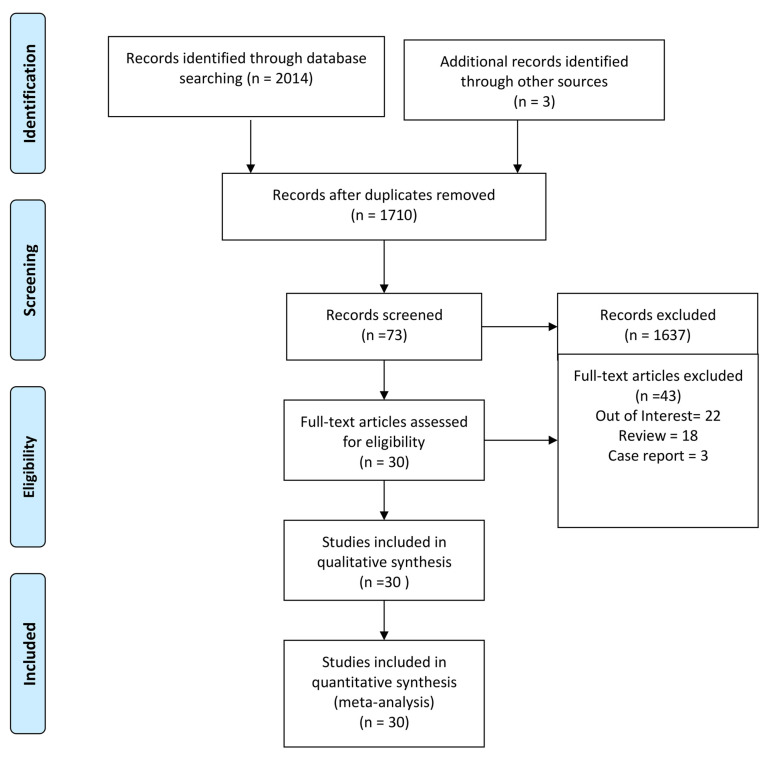
PRISMA 2009 flow diagram [8].

In the review, we incorporated all the peer-reviewed published papers reporting data on lung involvement in pSS patients. We included all the works conducted in pSS patients (Population) with lung involvement evaluation (Intervention and Control) that reported ILD prevalence (Outcome). No time limit on study publications was set during research. We conducted a systematic search in MedLine (via PubMed), Embase and Cochrane databases up to 7 December 2022. The main search was conducted using the string (“interstitial lung disease” OR “ILD pattern” OR “ILD” OR “pulmonary involvement” OR “progressive fibrosis” OR “pulmonary fibrosis” OR “active pulmonary involvement” OR “fibrosis” OR “lung involvement”) AND (“Sjogren’s” OR “Sjogren’s syndrome” OR “sjogren syndrome” OR “Sjogren” OR “primary sjogren’s syndrome” OR “pss” OR “sjs”). In addition, relevant keywords were used in different combinations for freehand search, and the bibliography of the selected articles was revised to improve the search strategy’s sensitivity, as shown in Figure 1.

Excluded from this study were review papers, case studies, correspondences, concise reports, non-English language publications, and those with missing data. The list of all excluded papers after the evaluation of the full text is provided in Supplementary Table S3.

### 2.3. Eligibility Criteria

For the primary search, based on preliminary scouting, we included all the clinical studies reporting results regarding the ILD prevalence in pSS patients.

### 2.4. Data Extraction and Quality Assessment

Data from the chosen articles were extracted, collected and summarized by three independent reviewers (AM, SDA, IG), and verified by two senior reviewers (OB, LN). From each selected article, the following features have been collected: first author; year of publication; origin; study design; total number of participants; age of participants; gender; methodology used to assess ILD; serological markers (anti-nuclear antibodies (ANA), anti-Ro (SSA), anti-La (SSB), C-reactive protein (CRP)), and carbon dioxide diffusing capacity corrected for haemoglobin concentration (DLco). The mean values of CRP, SSA, SSB, age of the participants, and DLco values were also extracted, when available. When summary statistics were not fully reported, these data were calculated whenever possible [10]. Where data were missing, incomplete or inconsistent, the authors were contacted to obtain necessary information.

### 2.5. Assessment of Methodological Quality

The quality of the studies was assessed using an adapted Assessment Tool for Prevalence Studies [11]. This tool evaluates the risk of bias in prevalence studies. It considers ten different items, such as the representativeness and the selection of the study population, the likelihood of non-response bias, the process of data collection, the appropriateness of the definition of cases (subjects with ILD), and the measurement of the parameter of interest (prevalence of ILD). The quality of the studies included in the quantitative analysis was assessed using the “star system” of the Newcastle–Ottawa Quality Assessment Scale (NOS) [12]. The score ranges from 0 to 9 stars (Supplementary Table S4). Studies that scored ≥7 stars were considered high quality. For case series studies, we evaluated the quality using the Quality Assessment Tool for Before-After (Pre-Post) Studies with No Control Group proposed by the National Heart, Lung, and Blood Institute—US Department of Health and Human Services (https://www.nhlbi.nih.gov/health-pro/guidelines/in-develop/cardiovascular-risk-reduction/tools/before-after, accessed on 1 December 2022). After scoring each item, an overall rate (good, fair, or poor) was assigned by each reviewer (Supplementary Table S5). Quality evaluation was performed independently by two reviewers (AM and IG). If there was any disagreement in the scores, a third reviewer (SDA) was involved to re-evaluate the original study.

### 2.6. Statistical Analysis

Analyses of data and graphs were performed using the package ‘metafor’ of the R statistical software (version 4.1.2, 2021; The R Foundation for Statistical Computing, Vienna, Austria). The pooled prevalence of ILD was estimated using a random-effects model. This model assumes that the included studies have varying effect sizes, thus providing a conservative estimate of the overall effect. The 95% confidence intervals (CIs) of the prevalence reported for each study were estimated from the proportion of cases and the specific sample size, using the binomial Clopper–Pearson exact method. Freeman–Tukey double arcsine transformation was applied to the primary study data to approximate normality. The final pooled results and 95% CIs were transformed and expressed as percentages for a simpler interpretation. An inverse variance method was used for weighting each study in the pooled estimates. We used Cochran’s Chi-square (Cochran’s Q) and I2 test to analyse the statistical heterogeneity between the results of different studies: I^2^ > 50% and/or *p* < 0.05 showed substantial heterogeneity [13]. An additional subgroup analysis was conducted, according to the diagnosis of ILD performing HRCT, to detect the possible source of the between-study heterogeneity. Sensitivity analyses were performed with the leave-one-out cross-validation test, by the sequential omission of individual studies to determine the contribution of each study to the pooled estimates, thus evaluating the stability and reliability of the results. Publication bias was explored through funnel plots [14] and the Begg adjusted rank correlation test [15]. To correct for publication bias, Duval and Tweedie’s ‘trim-and-fill’ analysis was carried out [16]. In the presence of an asymmetric funnel shape, this test detects putative missing studies to rebalance the distribution and provides an adjusted pooled estimate taking the additional studies into account, thus correcting the analysis for publication bias. Available covariates that could affect the estimates, such as publication year, study design, geographic region, and mean values of CRP, anti-SSA antibodies, anti-SSB antibodies, and DLco values of the study populations, were included in linear meta-regression models.

## 3. Results

### 3.1. Study Selection and Characteristics

Using the search strategy, 2017 peer-reviewed articles were retrieved. After the first scrutiny checking titles and abstracts, 73 articles were selected for full-text assessment. After review, 30 studies were included in the qualitative and quantitative analysis. Eleven studies had a prospective design, and among them four were conducted in Greece [17,18,19,20], three in Sweden [21,22,23], one in Spain [24], one in the Netherlands [25], two in Italy [26,27], and one in China [28]. Nineteen studies had a retrospective design, and among them ten were conducted in China [28,29,30,31,32,33,34,35,36,37], three in Turkey [38,39,40], two in France [41,42], one in Italy [26], one in the Netherlands [43], one in Japan [44], one in Spain [45], and one in Germany [46]. Many of them referred to pSS patients fulfilling the revised criteria proposed by the American–European Consensus Group [47]. In 23 studies out of the 30 included HRCT was performed to investigate lung involvement [19,20,25,26,27,28,29,30,31,32,33,35,36,37,38,39,40,41,42,43,44,45,46]. The main characteristics of the selected studies are reported in Table 1. The overall quality of the selected studies is high, although studies with a control group have a lower quality (Tables S4 and S5).

### 3.2. ILD Prevalence in Sjogren’s Syndrome

**Figure 2:**
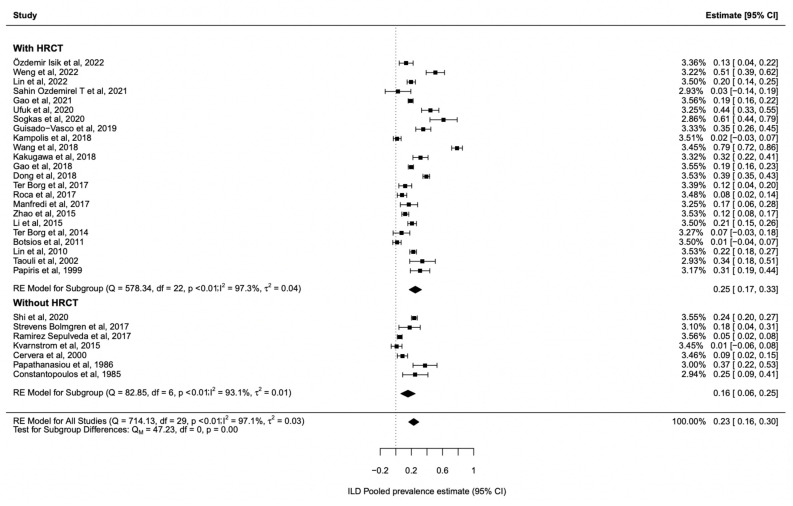
Forest plots depicting the pooled prevalence estimate for ILD in Sjogren patients with subgroup analyses for HRCT. Diamonds indicate the overall summary estimates, and width of the diamonds represents the 95% confidence interval (CI); boxes indicate the weight of individual studies in the pooled results [17,18,19,21,22,23,24,25,26,27,28,29,30,31,32,33,34,35,36,37,38,39,40,41,42,43,44,45,46].

**Figure 3:**
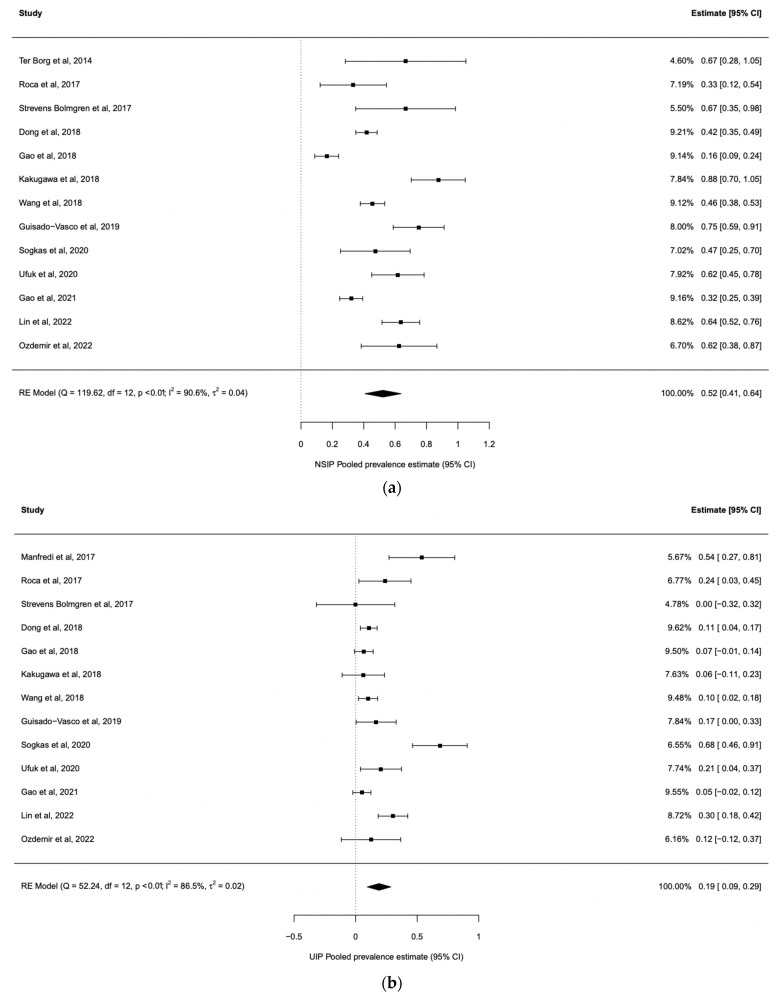
Forest plots depicting the pooled prevalence estimate for NSIP and UIP in ILD patients. (**a**) Forest plots depicting the pooled prevalence estimate for NSIP in ILD patients. Diamonds indicate the overall summary estimates, and width of the diamonds represents the 95% confidence interval (CI); boxes indicate the weight of individual studies in the pooled results. (**b**) Forest plots depicting the pooled prevalence estimate for UIP in ILD patients. Diamonds indicate the overall summary estimates, and width of the diamonds represents the 95% confidence interval (CI); boxes indicate the weight of individual studies in the pooled results [17,18,19,21,22,23,24,25,26,27,28,29,30,31,32,33,34,35,36,37,38,39,40,41,42,43,44,45,46].

**Figure 4:**
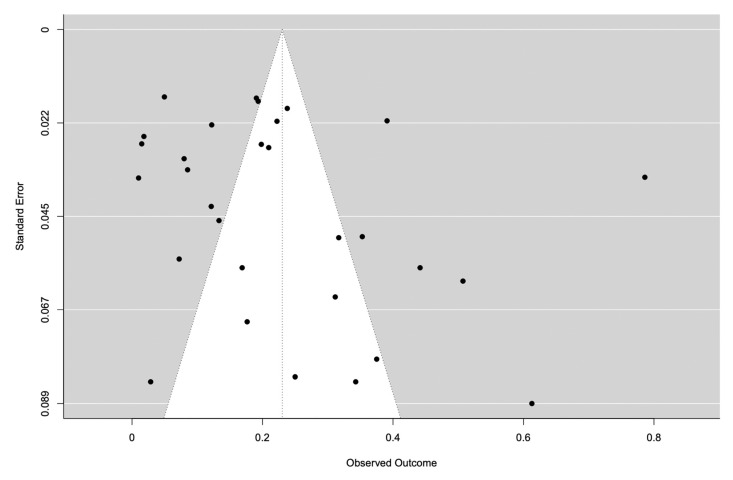
Contour-enhanced funnel plot. The funnel is centred at 0. Various levels of statistical significance of the points, representative of the studies, are indicated in the plot.

We analysed the pSS-ILD prevalence in 30 studies with 1557 pSS-ILD patients among 8255 pSS patients (Table 1). The prevalence of pSS-ILD in the selected studies ranged from 1% to 75%, and the pooled pSS-ILD prevalence was 23% (95% CI: 16–30) (Figure 2) with a prominent heterogeneity (*I*^2^ = 97%) (Table 1). We also explored the role of HRCT to better define the presence of ILD in these patients, performing a subgroup analysis. Our results show that the use of HRCT is associated with a significant higher prevalence of pSS-ILD (25%, 95% CI 17–33) when compared with the prevalence of pSS-ILD diagnosed by using PTFs (16%, 95% CI 6–25) (*p* < 0.001), as shown in Figure 2. Furthermore, we evaluated the pooled prevalence of the two main clinical radiological patterns, NSIP and UIP, in these patients. As far as the NSIP prevalence was concerned, we retrieved 13 papers [25,28,32,33,35,36,38,40,42,44,45,46] including 3599 patients with a pooled prevalence of 52% (95% CI: 41–64), with a high heterogeneity I^2^ = 90.6% (Figure 3a). On the other hand, UIP prevalence was reported in 13 studies [26,28,32,33,35,36,38,40,42,44,45,46] including 3621 patients with a pooled prevalence of 44% (95% CI: 32–55, I^2^ = 91%) (Figure 3b). Publication bias was evaluated with the funnel plot. The Egger’s test was used to test funnel plot asymmetry, and the analysis showed t = 1.7700, *p* = 0.077, not suggesting asymmetry (Figure 4). The “leave-one-out” test did not identify a single study which could influence the estimate overall effect-size (Supplementary Table S6). By this methodology, *p*-values were always <0.0001.

### 3.3. Risk Factors Associated with pSS-ILD

A meta-regression analysis was conducted to test whether part of the heterogeneity might be due to the influence of moderators. ANA positivity, antiRo-SSA positivity, and antiLa-SSB positivity did not significantly contribute to the observed heterogeneity. On the other hand, male gender, DLco value, country, and HRCT seemed to significantly contribute to the observed heterogeneity, as listed in Table 2.

## 4. Discussion

pSS is usually described as a disease with a low mortality risk, except for the development of lymphoma, but in recent years the presence of lung involvement and ILD has been described as organ complications associated with an increased risk of death, with RR 2.54 (95% CI:1.28, 5.04) [48]. These data account for a new interest concerning the ILD prevalence in pSS, and consequently for the better management of the lung involvement associated with this disease. In this study, we performed an SLR and meta-analysis of all the available studies for ILD evaluation in patients with pSS and, of note, it is the first paper evaluating the impact of HRCT in defining in a more accurate way the ILD prevalence in these patients. By this strategy, our results show a pooled pSS-ILD prevalence of 23% (95% CI: 16–30), which is higher than the prevalence reported in another study, which showed a pooled prevalence of 13% (95% CI: 9–19) [49]. Compared to this previous meta-analysis, performed more than 5 years ago, we found nine new papers published in recent years exploring the pSS-ILD prevalence [34,35,36,37,38,39,40,45,46]. These data confirm, on one hand, that pSS-ILD is an emerging hot topic, and on the other hand the need for “refreshing” previous metanalyses considering the relatively fast increase of knowledge in the field of autoimmune diseases. Among the papers published in recent years, only one study did not use HRCT for the ILD diagnosis [34]. This observation confirms the increased importance of HRCT to diagnose ILD, in contrast to what has been used in past years, in which many studies defined ILD only by PFTs, thus missing a substantial number of patients. In fact, the use of HRCT for ILD assessment is associated with a higher ILD prevalence, as shown in Figure 2, where studies with HRCT had a higher ILD prevalence when compared to the others (25%, 95% CI 17–33 vs. 16%, 95% CI: 6–25, *p* < 0.001). These data mirror what has already been described in systemic sclerosis (SSc): HRCT improves the sensitivity for ILD diagnosis, when compared to the other tests such as PFTs [50]. In fact, data on SSc showed that 90% of SSc patients had interstitial abnormalities detected by HRCT, while only a percentage ranging from 40 to 75% of them had changes detectable by PFTs [51]. Further, we explored the pooled prevalence of NSIP and UIP in our patients to define the most common ILD radiologic pattern associated with pSS. Our study shows that NSIP is the most frequent pattern, with a pooled prevalence of 52% (CI: 41–64), while UIP pattern was detected in 44% (CI: 32–55) of the patients enrolled in the studies (Figure 3). Data regarding other patterns, such as LIP, were scarce. Our results resemble what was already observed in other systemic autoimmune disease, such as SSc and systemic lupus erythematosus, and suggest that different pathogenic mechanisms associated with specific autoimmune diseases may finally lead to common radiologic changes [52,53]. When we explored the factors associated with pSS-ILD, male gender, DLco value, country, and HRCT were associated with a higher pSS-ILD prevalence. Unfortunately, data retrieved from the available literature did not allow us to evaluate whether any of these moderators may correlate with a specific radiologic pattern. According to our work, mirroring a previous meta-analysis, male gender is significantly associated with pSS-ILD prevalence [49]. A different clinical phenotype in male pSS patients when compared to female pSS patients has already been shown [54]. Male patients have a higher frequency of lymphoma and an increased prevalence of serum anti-La/SSB antibodies when compared to females [54,55]. A decrease in DLco, although not specific, is a suitable and reliable clinical biomarker associated with ILD progression in patients with connective tissue diseases, thus supporting its role in the follow-up of the patients [56]. As far as the association with the geographical localization of the patients is concerned, many data support the role of geographic and ethnic backgrounds in determining the clinical phenotype in pSS patients [57]. Furthermore, a previous meta-analysis showed a significantly higher pSS-ILD prevalence in Asia than in Europe [49]. To observe the role of ethnicity, Brito-Zeron et al. analysed data from 9352 pSS patients living in Europe but with different origins. They found a significant different distribution of ILD in pSS patients with different ethnicities, with a lower distribution in the Hispanic group when compared to White, Black/African American, and Asian patients [57].

We did not find any association between the serologic status in terms of autoantibodies presence and the ILD prevalence. We know that anti-Ro/SSA antibodies have a pivotal role in pSS classification and mirror what happens in the salivary glands, and their presence is associated with early diagnoses, parotidomegaly, and sicca symptoms [58,59,60]. Data regarding the association between anti-Ro/SSA antibodies presence and pSS-ILD are controversial, and their relation should be clarified [32,61,62].

We are aware of some weakness of our paper, deriving from the small number of prospective studies published in the available literature, the heterogeneity of the study design, the small number of patients enrolled in the studies, and the variability in ILD definition. These differences partially explain the large variability observed in the results. On the other hand, ILD is now considered one of the most common and severe morbidities in many immunologic diseases, both autoimmune and autoinflammatory, sometimes being the main cause of death in these patients [58,59,60,63,64,65]. More data are needed to better explore ILD prevalence, and to clarify whether the observed differences may be related to the studies’ characteristics. Alternatively, it may be possible that different clinical and biological phenotypes may be associated with a different pulmonary involvement.

To date, no drug is specifically approved for pSS treatment. Guidelines for ILD treatment during pSS should be related to the extension and progression of the pulmonary involvement and, borrowing from experiences in other immune mediated diseases, glucocorticoids or other immunosuppressive drugs should be used [66]. Data deriving from studies specifically designed in pSS-ILD are still a strong unmet need, especially with the advent of anti-fibrotic drugs, and for these reasons it is impossible to derive any generalization.

In our paper, as far as the pSS-ILD prevalence is concerned, a large variability was observed and a previous metanalysis, published five years ago, may be considered old due to the velocity of progress in medical science. The methodology applied to our SLR highlights the differences among all the studies exploring the pulmonary involvement in pSS, such as the study design, the patients’ characteristics and 4eselection, the different geographical areas, and the different diagnostic methods. This heterogeneity confirms the need for specifically designed studies to assess the lung involvement in pSS patients, using well-defined and homogeneous criteria of inclusion, aimed at providing firm conclusions.

In conclusion, our data show that at least 20% of pSS patients have a comorbid ILD, usually the NSIP radiologic pattern. Male gender and the decrease in DLco value may be considered the most important independent factors supporting an active search of lung complications during the clinical history of pSS patients.

## Figures and Tables

**Table 1:** Main characteristics of included studies.

Study	Design	Country	n	pSS Classification Criteria	F, %	ILD, n (%)	HRCT	NSIP, n (%)	UIP, n (%)	PFTs	DLCO (mmol/min kPa)
Constantopoulos et al., 1985 [17]	Prospective	Greece	36	*	91.7%	9 (25%)	-	-	-	-	
Papathanasiou et al., 1986 [18]	Prospective	Greece	40	*	100%	15 (37.5%)	-	-	-	yes	91.23 ± 18.86
Papiris et al., 1999 [19]	Prospective	Greece	61	1993 ECCC	95%	19 (31%)	yes	-	-	yes	85 ± 17.7
Cervera et al., 2000 [24]	Prospective	Spain	223	1993 ECCC	91.5%	19 (8.5%)	-	-	-	-	
Taouli et al., 2002 [41]	Retrospective	France	35	1993 ECCC	80%	12 (34.3%)	yes			yes	71.9 ± 15.7
Lin et al., 2010 [29]	Retrospective	China	522	2002 AECG	91%	116 (22.2%)	yes	-	-	-	-
Botsios et al., 2011 [27]	Retrospective	Italy	336	2002 AECG	96%	5 (1.5%)	yes	-	-	-	-
Ter Borg et al., 2014 [25]	Prospective	Netherlands	83	2002 AECG	89%	6 (7.2%)	yes	4 (4.8%)		-	-
Kvarnstrom et al., 2015 [21]	Prospective	Sweden	199	2002 AECG	93%	2 (1%)	-	-	-	-	-
Li et al., 2015 [30]	Retrospective	China	315	2002 AECG	96%	66 (21%)	yes			yes	-
Zhao et al., 2015 [31]	Retrospective	China	483	2002 AECG	94%	59 (12.2%)	yes	-	-	-	-
Manfredi et al., 2017 [26]	Prospective	Italy	77	2002 AECG	88%	13 (16.9%)	yes	-	7 (9%)	yes	-
Ramirez Sepulveda et al., 2017 [22]	Prospective	Sweden/Norway	967	2002 AECG	93%	48 (5%)	-	-	-	-	-
Roca et al., 2017 [42]	Retrospective	France	263	2002 AECG	-	21 (8%)	yes	*7* (33.3%)	5 (23.8%)	yes	
Strevens Bolmgren et al., 2017 [23]	Prospective	Sweden	51	2002 AECG	96%	9 (17.6%)	-	6 (11.7%)	0		6.5 ± 1.9
Ter Borg et al., 2017 [43]	Retrospective	Netherlands	140	2002 AECG	89%	17 (12.1%)	yes	-	-	-	-
Dong et al., 2018 [32]	Retrospective	China	527	2002 AECG/2016 ACR EULAR	88%	206 (39%)	yes	86 (41.7%)	22 (10.7%)	yes	54.54 ± 21.25
Gao et al., 2018 [33]	Retrospective	China	853	2002 AECG	-	165 (31.7%)	yes	27 (39.1%)	11 (15.9%)	yes	57.5 ± 21.2
Kakugawa et al., 2018 [44]	Retrospective	Japan	101	2002 AECG	94%	32 (1.8%)	yes	28 (27.7%)	2 (2.0)	-	-
Wang et al., 2018 [28]	Prospective	China	201	2002 AECG	88%	158 (78.6%)	yes	72 (45.5%)	16 (10.1%)	yes	42.9 ± 19.4
Kampolis et al., 2018 [20]	Prospective	Greece	384	2002 AECG	94.5%	7 (1.8%)	yes	-	-	yes	81.74 ± 17.38
Guisado-Vasco et al., 2019 [45]	Retrospective	Spain	102	2016 ACR/EULAR	93%	36 (35.3%)	yes	27 (26%)	6 (5.9%)	yes	-
Sogkas et al., 2020 [46]	Retrospective	Germany	31	2016 ACR/EULAR	71%	19 (61%)	yes	9 (29%)	13 (42%)	yes	-
Shi et al., 2020 [34]	Retrospective	China	706	2002 AECG	90.5%	168 (23.8%)	-	-	-	-	-
Ufuk et al., 2020 [38]	Retrospective	Turkey	28	2016 ACR/EULAR	86%	34 (75%)	yes	21 (75%)	6 (21.4%)	yes	
Gao et al., 2021 [35]	Retrospective	China	934	2002 AECG	-	178 (19%)	yes	57 (44.9%)	9 (15.0%)	yes	72.4 ± 20.9
Sahin Ozdemirel et al., 2021 [39]	Retrospective	Turkey	35	2016 ACR/EULAR	94%	1 (3%)	yes	-	-	yes	91.28 ± 19.70
Lin et al., 2022 [36]	Retrospective	China	333	2002 AECG	93.1%	66 (19.8%)	yes	42 (63.6%)	20 (30.3%)	yes	58.82 ± 21.04
Weng et al., 2022 [37]	Retrospective	China	69	2016 ACR/EULAR	90%	35 (50%)	yes	-	-	yes	-
Işik et al., 2022 [40]	Retrospective	Turkey	120	2016 ACR/EULAR	-	16 (13.3%)	yes	10 (62.5%)	2 (12.5%)	yes	60.1 ± 20.4

* Other criteria before 2002 AECG: xerostomia, sicca syndrome, focal lymphocyte infiltrate on minor salivary gland biopsy. *n*, number of patients; ILD, interstitial lung disease, UIP, usual interstitial pneumonia, NSIP, non-specific interstitial pneumonia; PFTs, pulmonary function tests. DLco results are presented as mean  ±  SD or as a percentage.

**Table 2:** Meta-regression analysis.

Moderator	Coefficient	SE	Z Value	*p* Value	95% CI
Male gender	0.24	0.044	5.54	<0.0001	0.16–0.33
ANA positivity	0.19	0.25	0.77	0.44	−0.30–0.68
Anti-Ro/SSa positivity	0.59	0.35	1.69	0.091	−0.096–1.28
Anti-La/SSb positivity	0.42	0.29	1.45	0.15	−0.15–0.98
DLCO value	0.47	0.17	2.68	0.0074	0.12–0.81
Country	0.18	0.045	4.02	<0.0001	0.092–0.27
HRCT	0.16	0.069	2.35	0.019	0.027–0.30

SE, standard error; CI, interval of confidence.

## Data Availability

All the data that have been used are reported in the article and Supplementary Materials.

## Supplementary Materials

The following supporting information can be downloaded at: https://www.mdpi.com/article/10.3390/jcm12072586/s1, Table S1: PRISMA checklist; Table S2: MOOSE (Meta-analyses Of Observational Studies in Epidemiology) Checklist; Table S3: Papers excluded from the analysis with the main reason; Table S4: Newcastle-Ottawa Assessment Scale for case-control studies; Table S5: Quality assessment of the included studies without the control group; Table S6: Leave-one-out test.
