# Supplementary material for: Interstitial Lung Disease and Pulmonary Damage in Primary Sjögren’s Syndrome: A Systematic Review and Meta-Analysis
Source: J Clin Med. 2023 Mar 29;12(7):2586. doi: 10.3390/jcm12072586 (PMC10095380; doi:10.3390/jcm12072586)
Supplement: Supplementary file 1 [file jcm-12-02586-s001.zip › jcm-2243477-supplementary.pdf]

Table S1. PRISMA checklist.

Section/topic	#	Checklist item	Reportedon page[#]
TITLE			
Title	1	Identify the report as a systematic review, meta-analysis, or both.	1
ABSTRACT			
Structured summary	2	Provide a structured summary including, as applicable: background; objectives; data sources; study eligibility criteria, participants, and interventions; study appraisal and synthesis methods; results; limitations; conclusions and implications of key findings; systematic review registration number.	1
INTRODUCTION			
Rationale	3	Describe the rationale for the review in the context of what is already known.	2
Objectives	4	Provide an explicit statement of questions being addressed with reference to participants, interventions, comparisons, outcomes, and study design (PICOS).	2-3
METHODS			
Protocol and registration	5	Indicate if a review protocol exists, if and where it can be accessed (e.g. web address), and, if available, provide registration information including registration number.	
Eligibility criteria	6	Specify study characteristics (e.g. PICOS, length of follow-up) and report characteristics (e.g. years considered, language, publication status) used as criteria for eligibility, giving rationale.	3
Information sources	7	Describe all information sources (e.g. databases with dates of coverage, contact with study authors to identify additional studies) in the search and date last searched.	3
Search	8	Present full electronic search strategy for at least one database, including any limits used, such that it could be repeated.	3
Study selection	9	State the process for selecting studies (i.e. screening, eligibility, included in systematic review, and, if applicable, included in the meta-analysis).	3
Data collection process	10	Describe method of data extraction from reports (e.g. piloted forms, independently, in duplicate) and any processes for obtaining and confirming data from investigators.	3
Data items	11	List and define all variables for which data were sought (e.g. PICOS, funding sources) and any assumptions and simplifications made.	4
Risk of bias in individual studies	12	Describe methods used for assessing risk of bias of individual studies (including specification of whether this was done at the study or outcome level), and how this information is to be used in any data synthesis.	4
Summary measures	13	State the principal summary measures (e.g. risk ratio, difference in means).	4
Synthesis of results	14	Describe the methods of handling data and combining results of studies, if done, including measures of consistency (e.g. I^2) for each meta-analysis.	4
Risk of bias across studies	15	Specify any assessment of risk of bias that may affect the cumulative evidence (e.g. publication bias, selective reporting within studies).	5
Additional analyses	16	Describe methods of additional analyses (e.g. sensitivity or subgroup analyses, meta-regression), if done, indicating which were pre-specified.	5
RESULTS			
Study selection	17	Give numbers of studies screened, assessed for eligibility, and included in the review, with reasons for exclusions at each stage, ideally with a flow diagram.	6
Study characteristics	18	For each study, present characteristics for which data were extracted (e.g. study size, PICOS, follow-up period) and provide the citations.	6
Risk of bias within studies	19	Present data on risk of bias of each study and, if available, any outcome level assessment (see item 12).	7
Results of individual studies	20	For all outcomes considered (benefits or harms), present, for each study: (a) simple summary data for each intervention group (b) effect estimates and confidence intervals, ideally with a forest plot.	7
Synthesis of results	21	Present results of each meta-analysis done, including confidence intervals and measures of consistency.	7
Risk of bias across studies	22	Present results of any assessment of risk of bias across studies (see Item 15).	7
Additional analysis	23	Give results of additional analyses, if done (e.g. sensitivity or subgroup analyses, meta-regression [see Item 16]).	8
DISCUSSION			
Summary of	24	Summarise the main findings including the strength of evidence for each main outcome;	8

evidence		consider their relevance to key groups (e.g. healthcare providers, users, and policy makers).	
Limitations	25	Discuss limitations at study and outcome level (e.g. risk of bias), and at review-level (e.g. incomplete retrieval of identified research, reporting bias).	8
Conclusions	26	Provide a general interpretation of the results in the context of other evidence, and implications for future research.	8
FUNDING			
Funding	27	Describe sources of funding for the systematic review and other support (e.g. supply of data); role of funders for the systematic review.	9

From: Moher D, Liberati A, Tetzlaff J, Altman DG, The PRISMA Group (2009): Preferred Reporting Items for Systematic Reviews and Meta-Analyses: The PRISMA statement. *PLoS Med* 6(7): e1000097. doi:10.1371/journal.pmed1000097.

Table S2. MOOSE (Meta-analyses Of Observational Studies in Epidemiology) Checklist.

Item No	Recommendation	Reported on Page No
	Reporting of background should include	
1	Problem definition	2
2	Hypothesis statement	2
3	Description of study outcome(s)	3
4	Type of exposure or intervention used	3
5	Type of study designs used	3
6	Study population	3
	Reporting of search strategy should include	
7	Qualifications of searchers (eg, librarians and investigators)	4
8	Search strategy, including time period included in the synthesis and key words	4
9	Effort to include all available studies, including contact with authors	4
10	Databases and registries searched	4
11	Search software used, name and version, including special features used (eg, explosion)	5
12	Use of hand searching (eg, reference lists of obtained articles)	4
13	List of citations located and those excluded, including justification	4
14	Method of addressing articles published in languages other than English	4
15	Method of handling abstracts and unpublished studies	4
16	Description of any contact with authors	1
	Reporting of methods should include	
17	Description of relevance or appropriateness of studies assembled for assessing the hypothesis to be tested	3-4
18	Rationale for the selection and coding of data (eg, sound clinical principles or convenience)	3-4
19	Documentation of how data were classified and coded (eg, multiple raters, blinding and interrater reliability)	3-4
20	Assessment of confounding (eg, comparability of cases and controls in studies where appropriate)	3-4
21	Assessment of study quality, including blinding of quality assessors, stratification or regression on possible predictors of study results	3-4
22	Assessment of heterogeneity	3-4
23	Description of statistical methods (eg, complete description of fixed or random effects models, justification of whether the chosen models account for predictors of study results, dose-response models, or cumulative meta-analysis) in sufficient detail to be replicated	3-4
24	Provision of appropriate tables and graphics	3-4
	Reporting of results should include	
25	Graphic summarizing individual study estimates and overall estimate	5
26	Table giving descriptive information for each study included	5
27	Results of sensitivity testing (eg, subgroup analysis)	5
28	Indication of statistical uncertainty of findings	5
Item No	Recommendation	Reported on Page No
	Reporting of discussion should include	
29	Quantitative assessment of bias (eg, publication bias)	8
30	Justification for exclusion (eg, exclusion of non-English language citations)	8
31	Assessment of quality of included studies	8
	Reporting of conclusions should include	
32	Consideration of alternative explanations for observed results	8
33	Generalization of the conclusions (ie, appropriate for the data presented and within the domain of the literature review)	8

34	Guidelines for future research	8
35	Disclosure of funding source	8

From: Stroup DF, Berlin JA, Morton SC, et al, for the Meta-analysis Of Observational Studies in Epidemiology (MOOSE) Group. Meta-analysis of Observational Studies in Epidemiology. A Proposal for Reporting. *JAMA*. 2000;283(15):2008-2012. doi: 10.1001/jama.283.15.2008.

Table S3. Papers excluded from the analysis with the main reason.

First author	Year	Title	Main reasonfor exclusion
Strimlan	1976	Pulmonary manifestations of Sjogren's syndrome	out of interest, no classification criteria listed, not primary sjogren's syndrome
Vitali C	1985	Lung involvement in Sjogren's syndrome: A comparison between patients with primary and with secondary syndrome	out of interest, no classification criteria listed, not only primary sjogren's syndrome, less than 30 patients
Hatron, P	1987	Subclinical lung inflammation in primary sjögren's syndrome. relationship between bronchoalveolar lavage cellular analysis findings and characteristics of the disease	out of interest, no classification criteria listed, less than 30 patients
Quismorio F	1996	Pulmonary involvement in primary Sjögren's syndrome	review
Cain HC	1998	Pulmonary manifestations of Sjögren's syndrome	review
Davidson B K	2000	Ten year follow up of pulmonary function in patients with primary Sjögren's syndrome	out of interest, concise report
Kim	2002	Interstitial lung diseases associated with collagen vascular diseases: Radiologic and histopathologic findings	out of interest, include patients with different autoimmune diseases
Kanoh, S	2003	Sjören's syndrome with infiltrative lung disease showing upper lung field predominance	case report
Parambil, J	2006	Interstitial lung disease in primary Sjögren syndrome	out of interest, less than 30 patients included
Parke A L	2008	Pulmonary manifestations of primary Sjögren's syndrome	review
Shi J	2009	Pulmonary manifestations of sjögren's syndrome	out of interest, less than 30 patients
Yazisiz, V	2010	Lung involvement in patients with primary Sjögren's syndrome: What are the predictors?	out of interest, only 14 patients with pulmonary involvement with not enough data
Nikpour, M	2010	Interstitial Lung Disease in Sjogrens Syndrome	review
Hatron, P	2011	Pulmonary manifestations of Sjögren's syndrome	review
Tomita, Y	2012	Rapidly progressive pulmonary fibrosis following the onset of diffuse alveolar hemorrhage in Sjögren's syndrome: An autopsy case report	case report
Stojan, G	2013	Pulmonary Manifestations of Sjögren's Syndrome	review
Palm, Ø	2013	Clinical pulmonary involvement in primary Sjögren's syndrome: Prevalence, quality of life and mortality - A retrospective study based on registry data	out of interest, the pulmonary involvement is not detailed
Yeh J	2014	Association between sjogren's syndrome and respiratory failure: Put airway, interstitia, and vessels close together: A national cohort study	out of interest, not enough data on pulmonary involvement
Kreider M	2014	Pulmonary involvement in Sjögren syndrome	review
Enomoto, Y	2014	Features of usual interstitial pneumonia in patients with primary Sjögren's syndrome compared with idiopathic pulmonary fibrosis	out of interest, comparison between UIP in pSS and IPF
Mira-Avendano, I	2015	Pulmonary manifestations of Sjögren syndrome, systemic lupus erythematosus, and mixed connective tissue disease	review
Flament, T	2016	Pulmonary manifestations of Sjögren's syndrome	review
Roca F	2017	Interstitial lung disease in primary Sjögren's syndrome.	out of interest,

		Review	The paper is listed as a
Vasco, P	2017	Assessment of interstitial lung disease in Sjögren’s syndrome by lung ultrasound: a pilot study of correlation with high-resolution chest tomography	out of interest. Less than 30 patients
Sebastian A	2017	Chest HRCT findings in patients with primary Sjögren's syndrome.	out of interest, data only in patients with pulmonary involvement
Strevens Bolmgren, V	2017	Respiratory symptoms are poor predictors of concomitant chronic obstructive pulmonary disease in patients with primary Sjögren’s syndrome	out of interest Only data on COPD are reported
McCoy, S	2017	Sjögren's syndrome-associated lung disease.	review
Lopez Velazquez, M	2018	Pulmonary manifestations of systemic lupus erythematosus and Sjögren’s syndrome	review
Jin, Y	2019	Clinical profile and associated factors of pulmonary involvement in primary Sjögren's syndrome	out of interest, not data on ILD and ILD pattern
Natalini, J	2019	Pulmonary Involvement in Sjögren Syndrome	review
Gupta, S	2019	Pulmonary manifestations of primary sjögren's syndrome: Underlying immunological mechanisms, clinical presentation, and management	review
Kamiya, Y	2019	Prognostic factors for primary Sjögren's syndrome-associated interstitial lung diseases	review
Chung, A	2019	Pulmonary and Bronchiolar Involvement in Sjogren's Syndrome	review
Gupta, S	2019	Pulmonary manifestations of primary sjögren's syndrome: Underlying immunological mechanisms, clinical presentation, and management.	review
Posso-Osorio, I	2019	Pulmonary involvement as the initial manifestation in primary Sjögren's syndrome	case report
Sambataro, D	2020	Patients with interstitial lung disease secondary to autoimmune diseases: How to recognize them?	review
Amlani, B	2020	Treatment of primary sjögren's syndrome-related interstitial lung disease: A retrospective cohort study	out of interest, only 19 patients
Heus, A.	2020	Pulmonary involvement in primary Sjögren’s syndrome, as measured by the ESSDAI.	out of interest, pulmonary involvement assumed to be related to pSS.
Alhamad, E	2021	Clinical characteristics and outcomes in patients with primary Sjogren's syndrome-associated interstitial lung disease	out of interest, patients selected for ILD presence
Manfredi A.	2021	Fibrosing interstitial lung disease in primary Sjogren syndrome	out of interest, patients selected for ILD presence
Peredo, R	2021	Sjogren’s Syndrome and Pulmonary Disease	review
Ottaviani, S.	2022	Rheumatological evaluation of patients with interstitial lung disease	out of interest, not specific for pSS

Table S4. Newcastle-Ottawa Assessment Scale for case-control studies.

Study	Selection				Comparability		Exposure			
	Definition of cases	Representativeness of cases	Selection of controls	Definition of controls	On age	On other risk factors	Assessment of exposure	Same methods of ascertainment for cases and controls	Non-response rate	Total score
- Taouli et al, 2002	★	★	★	★	☆	★	★	★	☆	7
- Lin et al, 2010	★	★	☆	☆	★	★	★	★	☆	6
- Botsios et al, 2011	★	★	★	★	★	★	★	★	☆	8
- Ter Borg et al, 2014	★	☆	☆	☆	★	★	☆	★	☆	5

- Kvarnstrom et al, 2015	★	☆	☆	☆	★	★	★	★	★	6
- Li et al, 2015	★	★	★	★	★	★	★	★	★	9
- Zhao et al, 2015	★	☆	☆	☆	☆	☆	★	★	★	6
- Manfredi et al, 2017	★	★	★	★	★	☆	★	★	★	8
- Gao et al, 2018	★	☆	★	★	★	★	★	☆	★	7
-Kakugawa et al, 2018	★	☆	★	★	★	★	★	☆	★	7
- Wang et al, 2018	★	☆	★	★	★	★	★	☆	★	7
- Kampolis et al, 2018	★	☆	★	★	★	★	★	☆	★	7
- Guisado-Vasco et al, 2019	★	☆	★	★	★	★	★	☆	★	7
- Sogkas et al, 2020	★	★	★	★	★	★	★	☆	★	8
- Shi et al, 2020	★	★	★	★	★	★	★	★	★	9
- Ufuk et al, 2020	★	★	★	★	★	★	★	★	★	9
- Sahin Ozdemirel T et al 2021	★	★	★	★	★	★	★	★	★	9
- Lin et al, 2022	★	★	★	★	★	★	★	★	★	9
- Weng et al, 2022	★	★	★	★	★	★	★	★	★	9
- Özdemir Işik et al, 2022	★	★	★	★	★	★	★	☆	★	8

Table S5. Quality assessment of the included studies without the control group.

Study	Quality Assessment	Item 1	Item 2	Item 3	Item 4	Item 5	Item 6	Item 7	Item 8	Item 9	Item 10	Item 11	Item 12
Constantopoulos et al, 1985	Poor	No	Yes	No	No	Not applicable	Not reported	No	No	Not reported	Yes	No	No
Papathanasiou et al, 1986	Poor	No	No	No	Not applicable	Not applicable	No	No	No	Not reported	No	No	No
Papiris et al, 1999	Poor	Yes	No	No	Not applicable	Not applicable	No	No	No	Not reported	Yes	Yes	No
Cervera et al, 2000	Fair	Yes	Yes	No	Yes	Not applicable	No	Yes	No	Not reported	Yes	Yes	No
Roca et al, 2017	Poor	Yes	No	No	No	Not applicable	No	No	No	Not reported	No	No	No
Strevens Bolmgren et al, 2017	Fair	No	Yes	No	No	Not applicable	Yes	Yes	No	Not reported	Yes	Yes	No
Ter Borg et al, 2017	Poor	Yes	No	No	Not applicabile	Not applicabile	No	No	No	Not reported	Yes	Yes	No
Dong et al, 2018	Fair	Yes	Yes	No	Yes	Not applicabile	No	No	No	Not reported	Yes	Yes	No

Quality Assessment Tool for Before-After (Pre-Post) Studies with No Control Group proposed by the National Heart, Lung, and Blood Institute - US Department of Health & Human Services (https://www.nhlbi.nih.gov/health-pro/guidelines/in-develop/cardiovascular-risk-reduction/tools/before-after).

Table S6. Leave-one-out test.

Study	Coefficent	SE	Z value	P value	95% CI
**Constantopoulos et al, 1985**	0.23	0.0345	6.65	<0.0001	0.16-0.29
**Papathanasiou et al, 1986**	0.22	0.0342	6.60	<0.0001	0.16-0.30
**Papiris et al, 1999**	0.24	0.0343	6.86	<0.0001	0.17-0.30
**Cervera et al, 2000**	0.24	0.0338	7.05	<0.0001	0.17-0.30
**Taouli et al, 2002**	0.24	0.0341	6.95	<0.0001	0.17-0.30
**Lin et al, 2010**	0.23	0.0346	6.71	<0.0001	0.17-0.30
**Botsios et al, 2011**	0.23	0.0348	6.62	<0.0001	0.16-0.30
**Ter Borg et al, 2014**	0.23	0.0345	6.60	<0.0001	0.16-0.30
**Kvarnstrom et al, 2015**	0.23	0.0343	6.60	<0.0001	0.16-0.29
**Li et al, 2015**	0.23	0.0348	6.64	<0.001	0.16-0.29
**Zhao et al, 2015**	0.24	0.0338	7.04	<0.0001	0.17-0.30
**Manfredi et al, 2017**	0.24	0.0342	6.88	<0.0001	0.17-0.30
**Ramirez Sepulveda et al, 2017**	0.23	0.0347	6.65	<0.0001	0.16-0.30
**Roca et al, 2017**	0.23	0.0345	6.79	<0.0001	0.17-0.30

**Strevens Bolmgren et al, 2017**	0.23	0.0346	6.72	<0.0001	0.16-0.30
**Ter Borg et al, 2017**	0.24	0.0343	6.87	<0.0001	0.17-0.30
**Dong et al, 2018**	0.23	0.0345	6.79	<0.0001	0.17-0.30
**Gao et al, 2018**	0.22	0.0342	6.56	<0.0001	0.16-0.29
**Kakugawa et al, 2018**	0.23	0.0347	6.67	<0.0001	0.16-0.30
**Wang et al, 2018**	0.23	0.0345	6.58	<0.0001	0.16-0.30
**Kampolis et al, 2018**	0.21	0.0274	7.58	<0.0001	0.15-0.26
**Guisado-Vasco et al, 2019**	0.24	0.0338	7.03	<0.0001	0.17-0.30
**Sogkas et al, 2020**	0.23	0.0344	6.57	<0.0001	0.16-0.29
**Shi et al, 2020**	0.22	0.0325	6.73	<0.0001	0.16-0.28
**Sahin Ozdemirel et al, 2021**	0.24	0.0340	6.95	<0.001	0.17-0.30
**Lin et al, 2022**	0.23	0.0347	6.67	<0.0001	0.16-0.30
**Weng et al, 2022**	0.22	0.0333	6.64	<0.0001	0.16-0.29
**Özdemir Işik et al, 2022**	0.23	0.0345	6.77	<0.0001	0.17-0.30
